# Comparison of Complications and Morbidity between Hand-Sewn and Stapled Anastomosis after Transhiatal Esophagectomy in Patients with Esophageal Cancer

**DOI:** 10.34172/mejdd.2025.432

**Published:** 2025-07-30

**Authors:** Parastou Shahryari, Mohsen Eshraghi, Seyed Jalal Eshagh Hosseini, Mostafa Vahedian, Farbod Eshraghi, Mohammad Hosein Atarod

**Affiliations:** ^1^Student Research Committee, Faculty of Medicine, Qom University of Medical Sciences, Qom, Iran; ^2^Department of Surgery, School of Medicine, Qom University of Medical Sciences, Qom, Iran; ^3^Department of Family and Community Medicine, School of Medicine, Spiritual Health Research Center, Qom University of Medical Sciences, Qom, Iran

**Keywords:** Esophageal cancer, Anastomosis, Stapling, Surgical complications, BMI, Readmission rates

## Abstract

**Background:**

With the increasing adoption of minimally invasive techniques in esophageal cancer (EC) surgery, comparing the complications and morbidity between hand-sewn anastomosis (HSA) and stapled anastomosis (SA) after transhiatal esophagectomy (THE) is of significant clinical importance. This study aimed to evaluate these two techniques in terms of postoperative complications, anastomotic leakage, hospital stay, and other related outcomes.

**Methods:**

This retrospective cohort study included 110 patients with EC who underwent THE at Shahid Beheshti Hospital. Patients were divided into two groups: those undergoing HSA and those undergoing SA. Demographic, surgical, and postoperative complication data were extracted from medical records and analyzed using SPSS software version 25. Quantitative variables were compared using the independent t-test, while qualitative variables were assessed using the chi-square or Fisher’s exact test. A *P* value<0.05 was considered statistically significant.

**Results:**

The results indicated that while the stapled method showed superiority in reducing recovery time and short-term complications, there was no significant difference in readmission rates between the two groups. This aligns with the findings of Law and colleagues, which suggest that readmission rates and disease recurrence are similar across different surgical methods.

**Conclusion:**

Both anastomosis techniques are comparable in terms of safety and potential for reoperation; however, further research is needed to investigate long-term complications. The findings of this study highlight the impact of body mass index and readmission rates on the choice of surgical method. Future research should address these discrepancies and include larger, more diverse populations in long-term assessments to achieve more definitive results regarding the comparative effectiveness of these techniques.

## Introduction

 In recent years, minimally invasive esophagectomy through the thoracic cavity has been gradually applied in the treatment of esophageal cancer (EC).^1-4^ Esophageal stripping and transhiatal esophagectomy (THE) have been developed to treat patients with EC with severe thoracic deformities or poor lung function who cannot tolerate thoracotomy.^5^ The comparison of complications and morbidity between hand-sewn anastomosis (HSA) and stapler anastomosis after THE is an important topic in surgical practice, particularly for patients with EC.^6-8^ The choice between hand-sewn and stapled anastomosis (SA) should be based on factors such as patient characteristics, the stage of cancer, surgeon experience, and institutional protocols. While some studies suggest that SA may result in fewer complications and morbidity, especially regarding anastomotic leakage, the decision must be individualized for each patient. Regarding the difference between these two methods, numerous studies and reports have been conducted worldwide. In most studies, less time, fewer suture leaks, shorter hospital stays, and lower mortality rates have been reported for surgical staples compared to sutures.^9^ Research comparing the complications and morbidity of hand-sewn and stapled anastomoses after THE surgery cannot only help improve the quality of surgical services but also lead to increased patient satisfaction and reduced healthcare costs. This study compares two primary techniques, HSA and SA, focusing on their associated morbidity, complication rates, and clinical implications.

## Materials and Methods

###  Study Design and Population

 This retrospective cohort study included patients with EC undergoing THE at Shahid Beheshti Hospital. Sampling was performed using a convenience sampling method. The required sample size for this study was calculated using a formula, considering a type I error rate of 5%, a power of 0.95, and standard deviations for the duration of surgery in transhiatal and thoracic surgeries of 35 and 54 minutes, respectively, with d = 46 based on similar studies.^10^ As a result, the sample size was determined to be 55 individuals in each group, leading to a total of 110 patients included in the study.

###  Inclusion/Exclusion Criteria

 The inclusion criteria for the study included all patients who underwent EC surgery at Shahid Beheshti Hospital during the study period. The exclusion criteria comprised incomplete files and patients who were unwilling to participate in the follow-up of the study. After obtaining approval from the Research Council of the Faculty of Medicine and the Code of Ethics from the Ethics Committee of Qom University of Medical Sciences (IR.MUQ.REC.1401.170), the researcher began collecting the necessary information for the study from patient files. This information included demographic details such as age, sex, underlying conditions, body mass index (BMI), imaging data, and surgical information about the patients, as well as other details like postoperative complications, disease recurrence, and the need for reoperation, which were recorded in a checklist.

###  Surgical Technique

####  Anastomosis Methods

 A uniform surgical team performed all procedures to minimize variability.

####  Hand-Sewn Anastomosis

- Technique: Single-layer or double-layer interrupted sutures using 3-0 or 4-0 absorbable monofilament sutures.
- Key steps: (1) Esophageal and gastric conduit mobilization. (2) End-to-side or end-to-end anastomosis under direct vision. (3) Interrupted sutures placed 1.5–2 mm apart, incorporating mucosa and muscularis.
- Surgeon experience: Performed by a single senior surgeon with > 10 years of experience in open esophageal surgery.

####  Stapled Anastomosis

- Device: Linear staplers for side-to-side anastomosis.
- Key steps: (1) Creation of parallel gastrotomy and esophagotomy. (2) Insertion of the stapler jaws into both lumens. (3) Firing of the stapler, followed by manual closure of the common enterotomy with 3-0 Vicryl sutures.
- Quality control: Intraoperative leak testing via air insufflation (20 cmH22​O pressure).

####  Postoperative Protocol

- Nasogastric tube removal on postoperative day 3.
- Contrast swallow study on day 7 to assess anastomotic integrity.

###  Data Analysis 

 Finally, the collected data were analyzed statistically using SPSS software version 25. To assess the normality of quantitative variables, the Kolmogorov-Smirnov test was used. Additionally, for comparing means between two groups, the independent t-test was employed, and for comparing qualitative variables, the chi-square tests and Fisher’s exact test were utilized. A significance level of less than 0.05 was considered.

## Results

 The average age of the patients was 64 ± 3 years. 67 patients (59.9%) were male and 43 patients (39.1%) were female. The demographic information of the patients was compared between the two groups, as shown in Table 1. There was no statistically significant difference in the mean age, sex, and the frequency of underlying diseases of the patients (*P* > 0.05). The frequency of BMI indicated that overweight was more prevalent in the stapler group, and a statistically significant difference was found in the frequency of BMI between the two patient groups (*P* < 0.05, Table 1).

**Table 1:** Comparison of demographic findings in patients undergoing hand-sewn and stapled anastomosis surgery

**Variable **		**Type of Anastomosis**		***P*** ** value**
		**Hand-sewn**	**Stapler**	
Age (mean ± SD)		64.4 ± 4.1	65.1 ± 3.4	0.775^*^
Sex (frequency, %)	Man	31 (28.2)	36 (32.7)	0.435^**^
	Women	24 (21.8)	19 (17.3)	
Underlying disease (frequency, %)	Yes	22 (20)	27 (24.5)	0.337^**^
	No	33 (30)	28 (25.5)	
BMI (frequency, %)	Thin	26 (23.6)	32 (29.1)	0.007^**^
	Normal	25 (22.7)	11 (10)	
	Overweight	4 (3.6)	12 (10.9)	

* t-test, **Chi-square.

 The average length of hospital stay, severity score, duration of surgery, healing time of the surgical site, and leakage at the surgical site were significantly lower in the stapled group than in the hand-sewn group (*P* < 0.05). There was only one case of death observed in the hand-sewn group. Additionally, the need for readmission did not show a statistically significant difference between the two groups (P > 0.05, Table 2).

**Table 2:** Comparison of clinical outcomes of hand-sewn and stapler anastomosis in surgical patients

**Variable **		**Type of anastomosis**		***P*** ** value**
		**Hand-sewn**	**Stapler**	
Hospitalization period (days, mean ± SD)		9.32 ± 1.87	3.65 ± 0.58	0.001^*^
VAS (scale: 0-10) (mean ± SD)		8.23 ± 0.5	6.52 ± 0.57	0.012^*^
Time of surgery (hours) (mean ± SD)		4.05 ± 0.49	3.74 ± 0.72	0.005^*^
Healing time of the surgical site (day) (mean ± SD)		9.3 ± 2.35	6 ± 0.63	0.001^*^
Need to refer again	Yes	41 (37.3)	32 (29.1)	0.069^**^
	No	14 (12.7)	23 (20.9)	
Death	Yes	1 (0.9)	0 (0)	0.998^**^
	No	54 (49.1)	55 (50)	
Leakage	Yes	17 (15.5)	0 (0)	0.001^**^
	No	38 (34.5)	55 (50)	

* t-test, **Chi-square.

## Discussion

 EC is the most common cancer in men and the third most common cancer among women in Iran. Therefore, conducting extensive studies and expert discussions on appropriate treatment methods seems essential. The present study aimed to determine and compare the complications and morbidity of two methods, hand-sewn and stapled anastomoses, following THE in patients with EC. The findings of our study highlight notable differences in clinical outcomes between stapled and HSA techniques. While demographic variables such as age, sex, and underlying diseases showed no significant intergroup differences, the stapled group exhibited a higher prevalence of overweight patients (10.9% vs. 3.6%, *P* = 0.007), which may reflect a potential selection bias or confounding factor in the distribution of BMI. Despite this, SA demonstrated clear short-term clinical advantages, including significantly shorter hospitalization periods (3.65 vs. 9.32 days, *P* = 0.001), reduced postoperative pain (VAS 6.52 vs. 8.23, *P* = 0.012), shorter operative times (3.74 vs. 4.05 hours, *P* = 0.005), and faster surgical site healing (6 vs. 9.3 days, *P* = 0.001). Most strikingly, anastomotic leakage, a critical complication, was absent in the stapled group, compared to 15.5% in the hand-sewn group (*P* = 0.001), underscoring the technical reliability of the stapler in minimizing this high-risk adverse event. However, readmission rates did not differ significantly between groups (*P* = 0.069), suggesting that while stapler anastomosis may reduce immediate postoperative morbidity (e.g., leakage), both techniques share comparable risks for complications requiring readmission. For instance, in our cohort, readmissions in the hand-sewn group were driven by leakage (15.5%), and delayed wound healing, whereas the stapled group experienced no leakage-related readmissions but had non-surgical causes (e.g., pneumonia). This aligns with prior studies indicating that stapler use improves procedural efficiency and acute outcomes but does not eliminate systemic or non-technical postoperative risks. The single mortality case in the hand-sewn group, though not statistically significant, warrants cautious interpretation as it may reflect broader trends of higher morbidity in manually sutured anastomoses. Nevertheless, the lack of significant differences in mortality and reoperation rates reinforces the overall safety of both methods. Another important finding in this study was the statistically significant difference in BMI between the two groups. Patients in the stapled group showed a higher prevalence of being overweight. This finding can contribute to a better understanding of how patients’ physical condition affects the choice of surgical method, suggesting that overweight patients may benefit more from the stapled technique. Hasegawa and colleagues conducted a study to investigate the negative impact of being overweight on short-term outcomes following esophagectomy for patients with esophageal squamous cell carcinoma (ESCC). They concluded that surgical treatment should not be denied to patients with ESCC due to overweight or underweight status. However, prevention during the procedure and close monitoring afterward for anastomotic leaks may be essential for overweight patients with ESCC following esophagectomy.^11^ Our results indicate that the average length of hospital stay, severity score, duration of surgery, and healing time of the surgical site were significantly lower in the stapler group compared to the hand-sewn group. These findings support the notion that the use of staplers can reduce surgical times and lead to faster patient recovery. Such benefits not only impact the quality of patient care but may also reduce hospital-associated costs related to prolonged patient stays. The significant reduction in hospital stay and recovery time observed in the stapler group is consistent with previous findings. For example, a meta-analysis conducted by Castro and colleagues analyzed various studies and showed that SA is associated with a significant reduction in hospital stay duration and faster recovery compared with hand-sewn methods. These results align with our findings and indicate that the use of a stapler may help expedite recovery.^12^ Our findings indicated that the rates of complications and mortality after surgery were low in the HSA group, particularly as only one case of mortality was observed. Studies, such as the one conducted by Liu and colleagues,^13^ also demonstrate that SA has a lower rate of leakage and complications compared to hand-sewn methods. However, some research, like that of Law and colleagues,^14^ reported no significant difference in leakage rates between the two methods, suggesting that while stapler techniques may have specific advantages, they cannot eliminate the risk of complications. These conflicting findings underscore the need for careful patient selection and consideration of individual risk factors. The lack of a significant difference between the two groups in our study is consistent with findings from Law and colleagues,^14^ which showed that readmission or recurrence rates following different surgical methods are comparable.

## Limitations and Suggestions

 In future studies, data analyses should be conducted across different BMI categories to determine the role of this variable. Additionally, limitations such as the small sample size and single-center design of the study reduce the generalizability of the results to broader populations. Although the stapler method has shown advantages in short-term recovery, its long-term outcomes—including the risk of stricture formation at the surgical site, complete functional recovery of organs, and cost-effectiveness compared with the hand-sewn method—remain underexplored and necessitate further investigation.

## Conclusion

 Overall, while our study’s findings support the benefits of SA in terms of recovery time and complications, there are specific variations regarding the impact of BMI and readmission rates. Future research should address these discrepancies and include larger and more diverse populations in long-term assessments to reach more definitive conclusions about the comparative effectiveness of these anastomosis techniques. By integrating our findings with existing studies, we can gain a better understanding of the intricacies of surgical methods in the context of EC treatment.
